# A Novel Modular Antigen Delivery System for Immuno Targeting of Human 6-sulfo LacNAc-Positive Blood Dendritic Cells (SlanDCs)

**DOI:** 10.1371/journal.pone.0016315

**Published:** 2011-01-21

**Authors:** Claudia C. Bippes, Anja Feldmann, Slava Stamova, Marc Cartellieri, Adrian Schwarzer, Rebekka Wehner, Marc Schmitz, E. Peter Rieber, Senming Zhao, Knut Schäkel, Achim Temme, R. Hal Scofield, Biji T. Kurien, Holger Bartsch, Michael Bachmann

**Affiliations:** 1 Institute of Immunology, Medical Faculty Carl Gustav Carus, Technical University Dresden, Dresden, Germany; 2 Center for Regenerative Therapies Dresden, Technical University Dresden, Dresden, Germany; 3 Third Hospital of Hebei Medical University, Hebei, China; 4 Oklahoma Medical Research Foundation (OMRF), Oklahoma City, Oklahoma, United States of America; National Institute of Virology, South Africa

## Abstract

**Background:**

Previously, we identified a major myeloid-derived proinflammatory subpopulation of human blood dendritic cells which we termed slanDCs (e.g. Schäkel et al. (2006) *Immunity* 24, 767–777). The slan epitope is an O-linked sugar modification (6-sulfo LacNAc, slan) of P-selectin glycoprotein ligand-1 (PSGL-1). As slanDCs can induce neoantigen-specific CD4+ T cells and tumor-reactive CD8+ cytotoxic T cells, they appear as promising targets for an *in vivo* delivery of antigens for vaccination. However, tools for delivery of antigens to slanDCs were not available until now. Moreover, it is unknown whether or not antigens delivered via the slan epitope can be taken up, properly processed and presented by slanDCs to T cells.

**Methodology/Principal Findings:**

Single chain fragment variables were prepared from presently available decavalent monoclonal anti-slan IgM antibodies but failed to bind to slanDCs. Therefore, a novel multivalent anti-slanDC scaffold was developed which consists of two components: (i) a single chain bispecific recombinant diabody (scBsDb) that is directed on the one hand to the slan epitope and on the other hand to a novel peptide epitope tag, and (ii) modular (antigen-containing) linker peptides that are flanked at both their termini with at least one peptide epitope tag. Delivery of a Tetanus Toxin-derived antigen to slanDCs via such a scBsDb/antigen scaffold allowed us to recall autologous Tetanus-specific memory T cells.

**Conclusions/Significance:**

In summary our data show that (i) the slan epitope can be used for delivery of antigens to this class of human-specific DCs, and (ii) antigens bound to the slan epitope can be taken up by slanDCs, processed and presented to T cells. Consequently, our novel modular scaffold system may be useful for the development of human vaccines.

## Introduction

B cells assemble a highly diverse repertoire of immunoglobulin heavy and light chains by recombination of variable (V), diversity (D), and joining (J) gene fragments, a process known as VDJ (heavy chain) or VJ (light chain) recombination e. g. [1]. Upon antigen encounter, antibody-encoding genes can undergo further modifications including somatic hypermutations (SHM) and class switch recombinations (CSR). CSR replaces the μ constant region (Cμ) in the heavy chain locus with one of the downstream constant regions (Cγ, Cε, Cα) leading to a switch from IgM type antibodies to antibodies of the IgG, IgE or IgA type [1]–[7]. After CSR and SHM, a positive selection favors the survival of those B cells which express high affinity antibodies (“affinity maturation”). Consequently, SHM and positive selection improve the affinity of the paratope(s) of an antibody towards its epitope.

Human and mouse antibodies of the IgM type are pentameric molecules containing ten paratopes. The binding avidity is the result of multiple cooperative binding interactions with the respective antigen. Most IgM antibodies have not undergone affinity maturation. Therefore, monovalently binding recombinant antibody fragments including for example single chain fragment variables (scFvs) prepared from IgM type antibodies usually retain only very low if any binding affinity e.g. [8].

In previous studies, the two IgM type monoclonal antibodies (mabs) MDC8 and DD2 were established and characterised [9]–[13]. Both mabs are directed to an O-linked carbohydrate modification (6-sulfo LacNAc) on PSGL-1 that is selectively expressed on a major population of myeloid derived human blood dendritic cells (DC). This population, formerly termed M-DC8+ DC, was recently renamed as slanDCs [12]. SlanDCs are a major population of proinflammatory human blood DCs, which mainly differ from other blood DC subsets by their selective phenotype (6-sulfo LacNAc+ ( =  slan+), CD1c−, CD11c+, CD14−, CD16+, CD45RA+, C5aR+) [9], [11], [12]. Functional data revealed that slanDCs efficiently induce neoantigen-specific CD4+ T cells and activate tumor-reactive CD8+ cytotoxic T cells (CTLs) [9], [11]. In addition, we found that slanDCs mediate antibody-dependent as well as -independent tumor-directed cytotoxicity and promote proliferation, IFN-γ production, and tumoricidal potential of NK cells [13].

Consequently, this subset of blood DCs represents a promising target for *in vivo* delivery of antigens for vaccination [12]. As mentioned above, all presently available anti-slanDC mabs (DD2 and MDC8) are IgM type antibodies. It is difficult to prepare a therapeutic drug from IgMs for treatment of humans according to the rules of good manufacturing practices (GMP) because one cannot safely remove potentially contaminating virus particles during purification. Here, we describe a novel modular multivalent anti-slan scaffold for delivery of antigens to slanDCs.

## Results

### Strategy for the development of an in vivo targeting system of slanDCs

**Figure 1:**
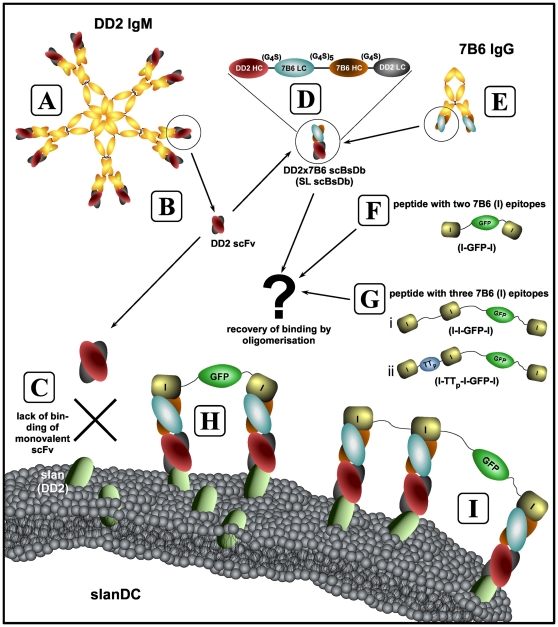
Recombinant antibodies derived from IgM antibodies: Restoring avidity by oligomerisation on a modular peptide scaffold. (A) The IgM anti-slan mab DD2 is a pentameric molecule. Preparation of a scFv (B) resulted in a monovalent molecule which failed to bind to slanDCs (C, data not shown). For recovery of binding avidity a scBsDb (D, SL scBsDb) and suitable linking peptide molecules (F,G) were constructed (H,I). In the scBsDb the variable heavy and light chain domains of the two mabs (A, DD2, S; E, 7B6, L) are recombinantly fused via glycine serine linkers (D; see also Fig. 3). This SL scBsDb is on the one hand directed to the slan epitope and on the other hand to a continuous peptide epitope (l-Tag). The linker peptide modules contain the l-Tag either two times (l-GFP-l, F) or three times (l-l-GFP-l, G(i); l-TT_p_-l-GFP-l, G(ii)). Binding of the SL scBsDb to the respective linker peptides results in the formation of a divalent (H) or trivalent (I) anti-slan scaffold with increased avidity.

In previous studies, we prepared and characterized the anti-slan mab DD2 [9]–[13]. As schematically summarised in Figure 1(A) this monoclonal IgM antibody has ten antigen binding sites. In a first step we generated a scFv from the anti-slan mab DD2 (Fig. 1B, DD2 scFv). In contrast to the polyvalent maternal IgM antibody, the recombinant monovalent scFv failed to bind to slanDCs (Fig. 1C, data not shown). We then tried to improve the affinity of the anti-DD2 slan scFv by introducing random mutations. In spite of a series of attempts, we were not able to isolate any DD2 derived mutant scFv which was capable to bind to slan DCs (data not shown). As schematically summarised in Figure 1 (D to I), in the next attempt we tried to partially restore the polyvalent anti-DD2 platform by oligomerisation of monovalent recombinant DD2 derived subunits: The idea was to create a scaffold consisting of a scBsDb and suitable linker peptides (Fig. 1H,I). As source for the scbsDb served two mabs including the anti-slan mab DD2 (Fig. 1D, DD2, S arm of the SL scBsDb) and a novel mab (Fig. 1E, 7B6, Bachmann unpublished; Fig. 1D, L arm of the SL scBsDb). The mab 7B6 is directed to the aa sequence EKEALKKIIEDQQESLNK which is part of the C-domain of a nuclear protein known as La protein [14]. We abbreviated this continous aa sequence as l-Tag. In addition to the SL scBsDb, linker peptide molecules (Fig. 1F,G) were constructed containing the l-tag either twice (Fig. 1F, l-GFP-l) or three times (Fig. 1G (i) l-l-GFP-l, (ii) l-TT_p_-l-GFP-l). We hoped that the formation of bi- (Fig. 1H), tri- (Fig. 1I), or other multivalent anti-slan scBsDb/linker peptide scaffolds would allow us to sufficiently restore a binding to slanDCs.

Until now it was also not known whether or not an antigen bound to the slan epitope will be taken up and delivered to the antigen presentation machinery of slanDCs. We, therefore, wanted to learn, whether or not (i) an experimental antigen can be delivered to slanDCs via such a scBsDb/linker peptide scaffold, and (ii) will be taken up by slanDCs, processed and presented to T cells. For this purpose, we planned to insert a common TT-derived T-cell epitope (TT_p_) between the first and the second linker epitope within the linker module l-l-GFP-l (Fig. 1G(ii)).

### Construction and expression of the SL scBsDb

**Figure 2:**
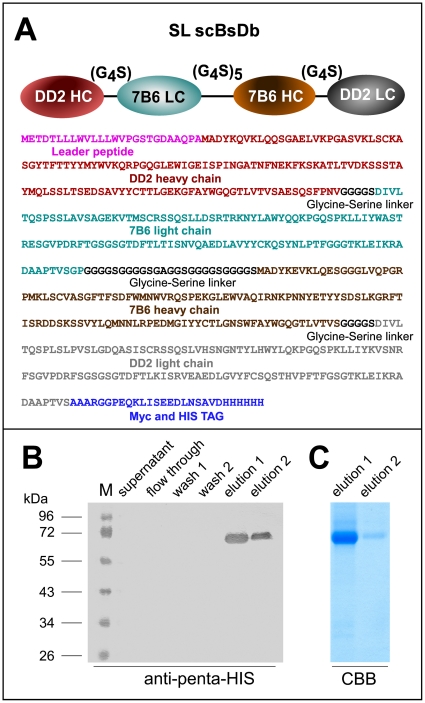
Construction and characterisation of the SL scBsDb. (A) A schematic view of the structure and the complete AA sequence of the SL scBsDb. (B) The SL scBsDb was expressed and purified as described under Materials and Methods. The flow through fractions, wash fractions (wash1, wash2) and the eluted fractions (elution 1, elution 2) containing the SL scBsDb were analyzed by SDS-PAGE (C) and immunoblotting (B). After SDS-PAGE separated protein(s) were stained with Coomassie brilliant blue (C, CBB), and after transfer the membrane was developed with an anti-penta-HIS antibody (B). M, marker proteins.

After amplification of the variable domains of the heavy and light chains of both mabs (DD2,7B6) they were fused via glycine-serine linkers as schematically shown in Figure 1D and Figure 2A. The cloning, eukaryotic expression and purification of the SL scBsDb is described in detail in Materials and Methods. Purified SL scBsDbs were analysed by SDS-PAGE (Fig. 2C) and immunoblotting (Fig. 2B). As shown in Figure 2 (B,C), the SL scBsDb could be efficiently expressed and purified by Ni-NTA affinity chromatography which is a prerequisite for a translation into the clinic.

### Construction and expression of multimeric linker peptides

**Figure 3:**
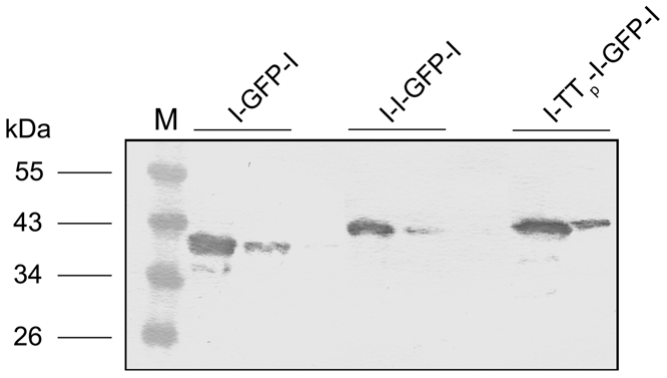
Characterisation of purified linker peptides. For the formation of bi- or trivalent anti-slan scaffolds peptide linker molecules were constructed containing the l-Tag twice (l-GFP-l) or three times (l-l-GFP-l; l-TT_p_-l-GFP-l). The purified peptide linkers were analysed by SDS-PAGE/immunoblotting using the anti-La mab 7B6 directed to the l-Tag. (M) Marker proteins.

Linker peptide modules were cloned according to Figure 1 (F,G) (see Materials and Methods), expressed in *E. coli* and purified. The purified proteins were analysed by SDS-PAGE/immunoblotting against the anti-La mab 7B6. As expected, the anti-La mab 7B6 reacted with the purified peptide linker proteins (Fig. 3) indicating that the l-tag(s) are present and accessible for the 7B6 paratope.

### Binding of the novel cell targeting system to the surface of slan(+) Jurkat cells

**Figure 4:**
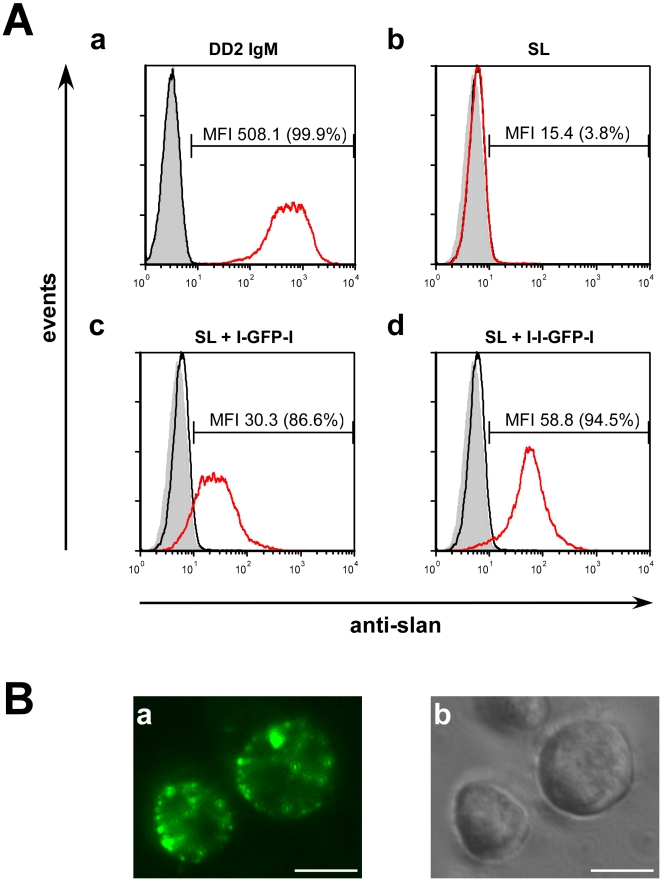
Analysis of binding of the SL ScBsDb/linker complex to DD2 positive Jurkat cells. (A), (a, black graph) IgM isotype negative control. (a, red graph) Binding of the anti-slan IgM mab DD2 to slan(+) positive Jurkat cells. (b, black graph) Anti-penta-HIS, isotype negative control. (b, red graph) Lack of binding of the monovalent SL scBsDb (SL) in the absence of a linker peptide. (c,d black graph) Isotype negative control. (c,d, red graph) Bivalent or trivalent anti-slan scaffolds consisting of the SL scBsDb and the linker peptide l-GFP-l (c, red graph) or l-l-GFP-l (d, red graph). (B), Detection of anti-slan scaffolds on the surface of Jurkat cells by epifluorescence microscopy. GFP (a), phase contrast (b), bar = 10 µm.

During previous studies e.g. [11] we had observed that a percentage of 5 to 10% of Jurkat cells are positive for the slan epitope. These slan epitope positive (slan(+)) Jurkat cells can be enriched by MACS (Materials and Methods) to a purity of more than 95% and kept in culture (Fig. 4A(a)). For estimation of the optimal formation and binding conditions of the complex, this Jurkat cell line was used instead of slanDCs. In agreement with previous data, the decavalent IgM anti-slan mab (DD2) is capable of staining slan(+) Jurkat cells (Fig. 4A(a, red graph)). In contrast, the monovalent anti-slan domain of the SL scBsDb fails to stain slan(+) Jurkat cells (Fig. 4A(b, red graph)). However, binding was regained when a multivalent anti-slan scaffold was formed by preincubation of the SL scBsDb with either the l-GFP-l (Fig. 4A(c, red graph)) or the l-l-GFP-l (Fig. 4A(d, red graph)) linker peptide. Using epifluorescence microscopy, we could also detect the GFP-labelled scBsDb/linker complexes on the surface of Jurkat cells (Fig. 4B(a,b)).

**Figure 5:**
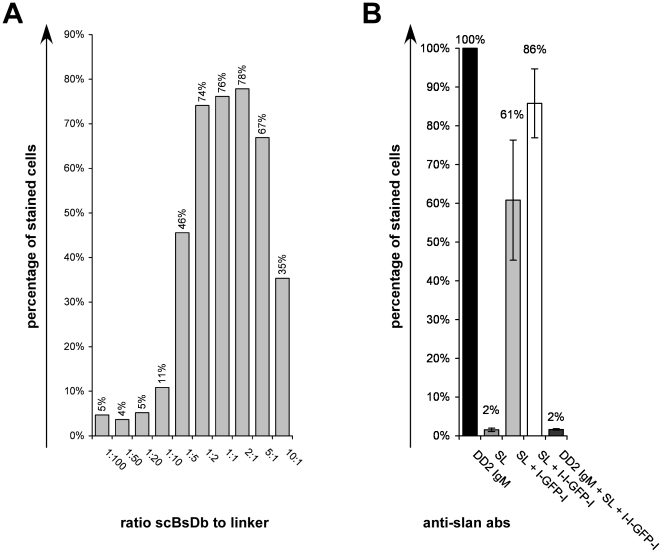
Binding of the SL scBsDb/linker complex to slan(+) Jurkat cells. (A) Anti-slan scaffolds were obtained by incubation of the SL scBsDb (SL) with the trivalent linker peptide l-l-GFP-l or the bivalent linker peptide (l-GFP-l, data not shown) at various ratios as indicated and analyzed for binding to slan(+) Jurkat cells. Binding of the maternal IgM anti-slan antibody DD2 was set as 100%. (B) After estimation of the optimal conditions, the binding of the bivalent (SL + l-GFP-l) and trivalent anti-slan scaffolds (SL+l-l-GFP-l) were compared to the binding of the maternal anti-slan IgM antibody DD2 (DD2 IgM). Moreover, the binding of the scaffolds in the presence of the maternal anti-slan IgM antibody DD2 was determined (DD2 IgM+SL+l-l-GFP-l).

In order to determine the optimal recovery conditions for binding, increasing amounts of the SL scBsDb (50, 100, 150, 200 pmol/50 µl) were preincubated with the l-l-GFP-l linker peptide (Fig. 5A) at molar ratios between 1 to 100 and 10 to 1. The formed complexes were incubated with slan(+) Jurkat cells and the binding was estimated by FACS analysis. Optimal bindings were seen at concentrations of 100 to 200 pmol/50 µl of the scBsDb (data not shown). The results for the combinations SL scBsDb (150 pmol/50 µl) and l-l-GFP-l linker peptide are shown in Figure 5 (A). The binding capability of the maternal IgM antibody was set to 100%. The optimal binding ratios were between 2 to 1 and 1 to 2. Using the l-l-GFP-l linker peptide we were able to recover between 78 and 95% of the maternal antibody binding (Figs. 4(d);5A,B). Using the l-GFP-l linker peptide the efficiency was less and ranged between 60 and 87% of the maternal antibody (Figs. 4(c);5B). The binding of the SL scBsDb/linker peptide scaffold depends on oligomerisation of the scBsDb via the linker peptide as 2 to 4% of the maternal antibody binding was achieved in the absence of a linker peptide (Fig. 4(b);5B, SL). Furthermore, preincubation of the anti-slan(+) Jurkat cells with the anti-slan IgM mab DD2 completely blocked the binding of the SL scBsDb/linker peptide scaffold (Fig. 5B, DD2 IgM+SL+l-l-GFP-l).

In summary, our data show that monovalent recombinant derivatives of the polyvalent anti-slan IgM mab DD2 are not capable of binding to slanDCs. However, this loss of binding can be restored by oligomerisation of monovalent anti-slan paratopes via the combination of SL scBsDbs with the peptide linker modules that contain two or more l-Tag epitopes. For restoring of the binding, a minimum of two anti-slan binding sites is required although three binding sites are clearly superior.

### Binding of the novel cell targeting system to the surface of slanDCs

**Figure 6:**
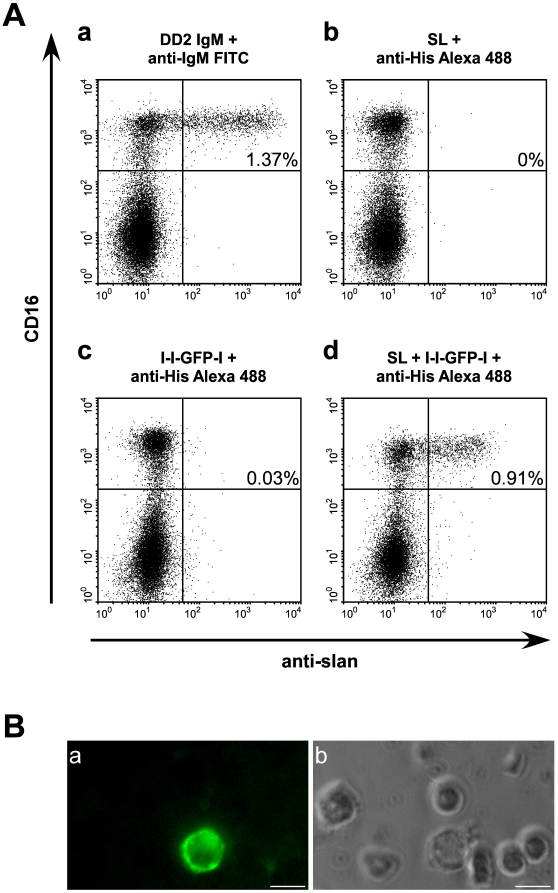
Binding of the SL scBsDb/linker complex to native slanDCs in PBMCs. (A) (a) Estimation of the amount of slan-DCs in a PBMC sample using the anti-slan IgM mab DD2. (d) Estimation of the amount of slanDCs in the same PBMC sample using the anti-slan scaffold consisting of the SL scBsDb and the l-l-GFP-l linker peptide. Control staining of the single components of the anti-slan scaffold including the SL scBsDb (b, SL) or the peptide linker module (c, l-l-GFP-l). (B) GFP-labelled anti-slan scaffolds identified by epifluorescence microscopy. GFP (a), phase contrast (b), bar = 10 µm.

Next, we analysed the binding capability and specificity of the SL scBsDb/linker peptide scaffold on human PBMCs (Fig. 6). As shown in Fig. 6A (a) the presented PMBC sample contained about 1.4% slanDCs, which is in a good agreement with previous estimations. As shown in Fig. 6A (d), the trivalent anti-slan scaffold consisting of the SL scBsDb and the l-l-GFP-l linker peptide allowed us to restore a specific immuno targeting of slanDCs in PBMCs. The single components of the scaffold system including the SL scBsDb (Fig. 6A(b)) and the l-l-GFP-l linker peptide (Fig. 6A(c)) did not bind to slanDCs. The presence of GFP in the linker module l-l-GFP-l allowed us to identify the SL scBsDb/linker peptide complexes on slanDCs by epifluorescence microscopy (Fig. 6B(a,b)). Similar results were obtained for PBMCs from three other human donors (data not shown).

### The presence of an experimental peptide antigen in a linker molecule does not interfere with the formation and binding of the multivalent anti-slan/linker peptide complex to slan(+) Jurkat cells

**Figure 7:**
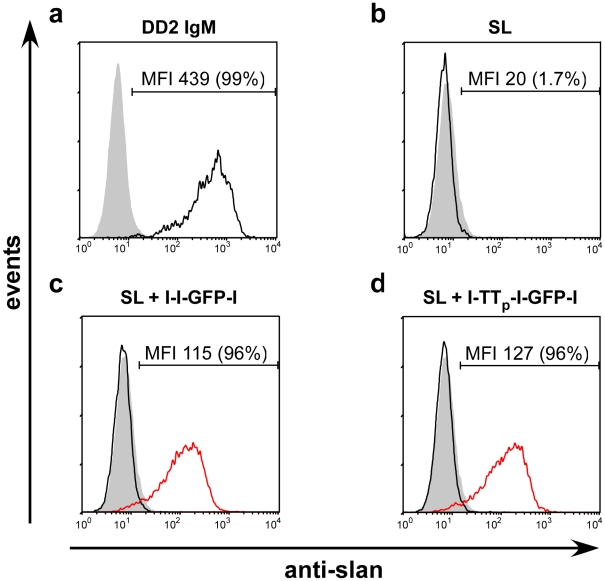
Immuno targeting of slan(+) Jurkat cells with a multivalent anti-slan scaffold containing a TT-derived T-cell epitope. (a, black graph) Binding of the anti-slan mab DD2 to slan(+) Jurkat cells. (c,d, red graph) Binding of anti-slan scaffolds consisting of the SL scBsDb and trivalent linker peptides either containing (c) or lacking (d) theTT-derived peptide epitope TT_p_. (b,c,d, black graph) Control binding of the single components of the anti-slan scaffolds including the SL scBsDb (b), the trivalent linker l-l-GFP-l (c), and the trivalent linker l- TT_p_ -l-GFP-l (d).

In order to provide first evidence for proof of principle of the novel immuno targeting strategy a known TT-derived T-cell epitope region (Materials and Methods, TT_p_) was fused in between the first and the second l-Tag of the l-l-GFP-l linker module (Fig. 1 G(ii)). As shown in Fig. 7(d, red graph) the complex formed between the SL scBsDb and this l-TT_p_-l-GFP-l linker peptide is capable of binding to slan(+) Jurkat cells. Moreover, the binding of the antigen-containing linker peptide/SL scBsDb complex is comparable to the binding of the l-l-GFP-l linker peptide/SL scBsDb complex (Fig. 7c, red graph). None of the used single components of the scaffold, including the SL scBsDb (Fig. 7(b, black graph)) or the linker peptide containing or lacking the TT derived peptide domain (Fig. 7(c,d, black graphs)), did bind on its own to slan(+) Jurkat cells.

Consequently, the presence of the TT-epitope in the peptide linker module does not interfere with the formation of the SL scBsDb/linker peptide scaffold and its binding to slan(+) cells.

### Stability of immune complexes present on the slan epitope of isolated slanDCs

**Figure 8:**
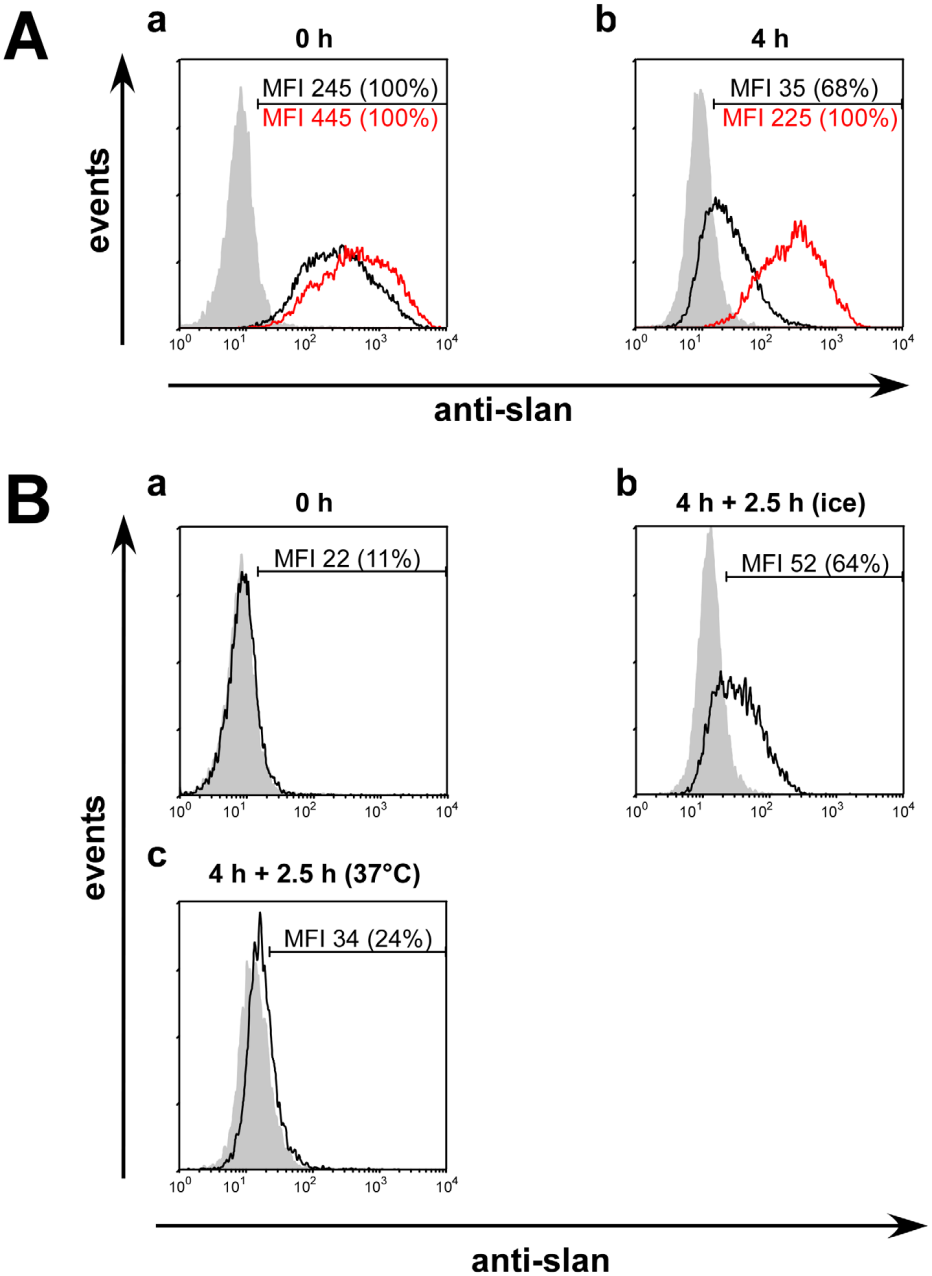
Stability of immune complexes formed via the slan epitope on isolated slanDCs. (A), (a, black graph) Staining of slanDCs freshly isolated by magnetic anti-IgM/MDC8 anti-slan beads with a secondary FITC-conjugated anti-mouse IgM antibody. (a, red graph) Freshly isolated slanDCs that are stained in addition with the anti-slan IgM mab DD2 and the secondary FITC-conjugated anti-mouse IgM antibody. (b, black graph) Staining of slanDCs with a secondary FITC-conjugated anti-mouse IgM antibody four hrs after isolation and an incubation at 37°C. (b, red graph) Staining of slanDCs with the anti-slan IgM mab DD2 and the secondary FITC-conjugated anti-mouse IgM antibody four hrs after isolation and an incubation at 37°C. (B), (a, black graph) Binding of the multivalent anti-slan scaffold is blocked to freshly isolated slan DCs. (b, black graph) Binding of the multivalent anti-slan scaffold consisting of the SL scBsDb (SL) and the l-TT_p_-l-GFP-l linker module regains four hrs after isolation and an incubation at 37°C. (c, black graph) Staining of slan DCs after an additional incubation of 2.5 h at 37°C. The scBsDb/peptide linker scaffold can no more be detected on the surface of slanDCs.

As described in Materials and Methods slanDCs can be isolated from PBMCs. For this purpose PBMCs were preincubated with the IgM anti-slan mab MDC8. SlanDCs were then isolated by anti-IgM immuno magnetic beads. As a consequence, such isolated slanDCs are covered with IgM anti-slan antibodies and anti-IgM beads and, therefore, almost all slanDCs are also stained by a FITC-labelled anti-IgM secondary antibody (Fig. 8A(a, black graph)). Thus, an additional staining by IgM anti-slan abs DD2 only increases the MFI values (Fig. 8A(a, red graph). After an incubation time of 4 h (Fig. 8A(b, black graph)) the FITC-conjugated secondary anti-IgM antibody stains only 68% of the slanDCs. This result indicates that the IgM abs and anti-IgM beads used for the isolation have partially disappeared from the surface of the slanDCs during this incubation time. This is also supported by the finding that both number and MFI of these slanDCs was increased when stained by the anti-slan IgM DD2 ab and the FITC-labelled secondary anti-IgM ab (Fig. 8A(b, red graph)). We interprete these data as follows: Anti-MDC8 IgM mabs and/or anti-MDC8/anti-IgM beads used for isolation of slanDCs are present on the surface of freshly isolated slanDCs and block the binding of the anti-slan mab DD2. Over time the MDC8/IgM immune complexes disappear from the surface and additional unbound slan epitopes become available on the cell surface which can then be stained by anti-slan (anti-DD2) abs. In agreement with this interpretation, SL scBsDb/linker module complexes also failed to bind on freshly isolated slanDCs (Fig. 8B(a, black graph)). However, the binding of this multivalent anti-slan scaffold consisting of the SL scBsDb and the l-TT_p_-l-GFP-l linker module recovers after the 4 hr-incubation period at 37°C (Fig. 8B(b, black graph)). After an additional incubation of 2.5 h at 37°C, the SL scBsDb/linker peptide complexes also disappear and can no more be detected on the surface of slanDCs (Fig. 8B(c, black graph)).

Taken together, these results indicate that (i) immune complexes bound to the slan epitope of PSGL-1 including immuno beads or scBsDb/linker peptide complexes are either taken up by slanDCs or released from the surface of slanDCs, and (ii) free “empty” slan epitopes reappear on the surface of slanDCs.

### Evidence for functional processing of the artificial antigen-containing linker peptides

So far our data only showed that the novel immuno targeting system can be used for the delivery of antigens to slanDCs in PBMCs. It still had to be shown that (i) anti-slan bound antigens can be taken up by slanDCs, and (ii) after uptake the antigens delivered via PSGL-1 will be processed and presented on MHC class II molecules to CD4+ T cells.

In case an antigen is taken up by a professional antigen presenting cell (APC) it will be processed and the resulting peptides will be presented on MHC class II molecules if fitting in the binding groove of the respective MHC class II molecule. If a T-cell epitope is obtained during cleavage and presented to memory CD4+ T cells this should lead to a T-cell proliferation. Hence, in the next step we wanted to show that T-cell epitopes when present in our novel artificial linker module will be released during processing and presented by APCs. As our l-TT_p_–l-GFP-l linker peptide contains artificial TT-derived T-cell epitope(s) we looked for healthy donors who were not only immunised with TT but whose T cells were also able to respond to our selected TT-derived peptide epitope.

**Figure 9:**
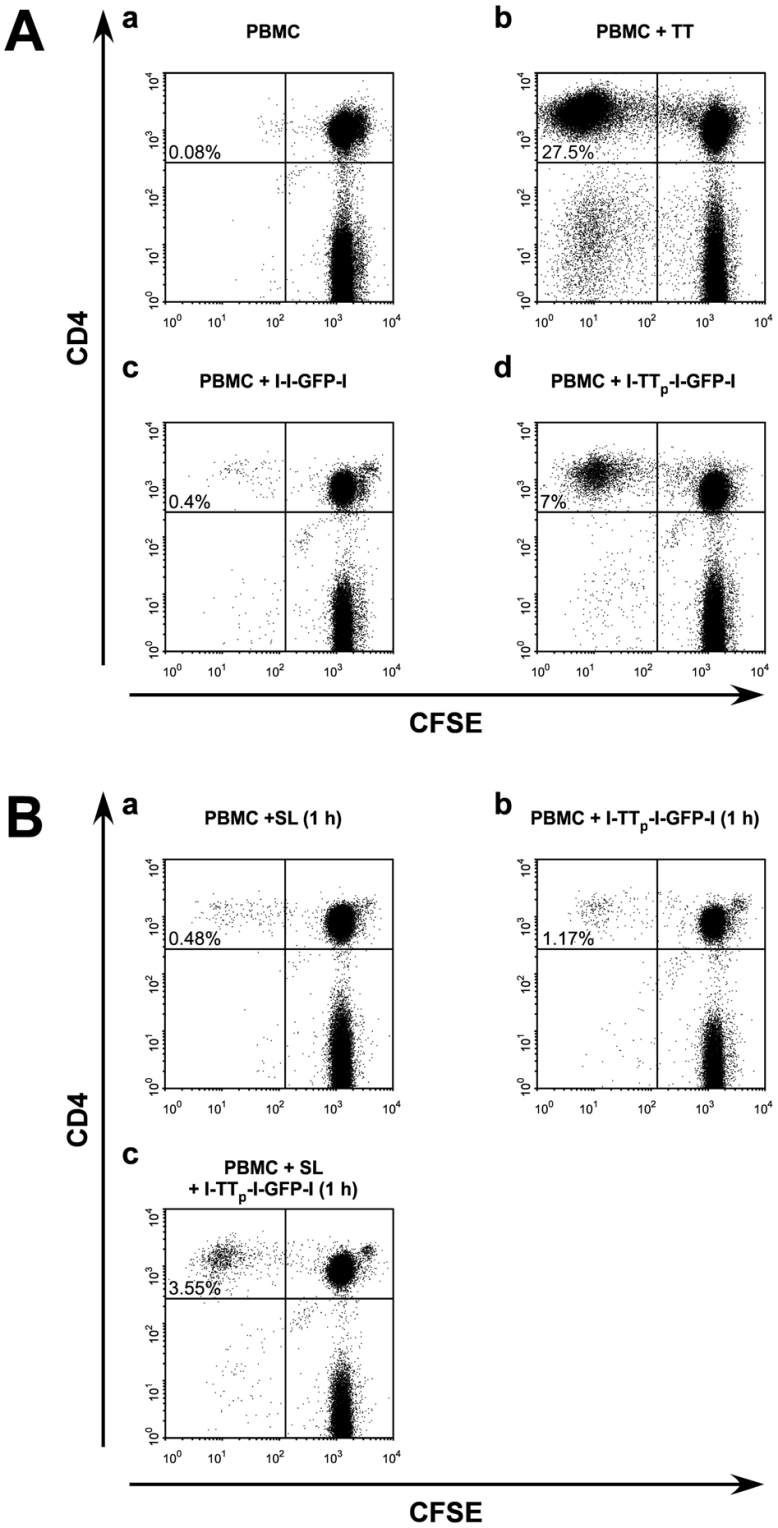
Analysis for proliferation of CD4+ T cells after delivery of the TT-derived T-cell epitope to slanDCs via the novel immuno targeting system. (A) CFSE labeled PBMCs were prepared and incubated at 37°C for eight days with either full length TT (b) or the linker module containing (d) or lacking (c) the TT-derived T-cell epitope TT_p_ and analysed by FACS. The data indicate that PBMCs of the selected donor contain anti-TT memory T cells that can be recalled by full length TT and to a less extent by the TT_p_ peptide linker but not by the linker peptide lacking the TT_p_ epitope. Untreated PBMCs (a). (B) CFSE labelled PBMCs were incubated with either the antigen-containing scaffold (c) or the single components (a,b) at 4°C. Unbound material was removed by washing.

The results for such a healthy donor are shown in Fig. 9 (A, (a to d)). PBMCs of this donor when incubated for eight days at 37°C with full length TT responded in a strong proliferation of CD4+ T cells (about 28%, Fig. 9A(b)). As expected a CD8 response for TT was very low (data not shown). In the absence of any recombinant protein, the T cells did not respond after the same incubation time (Fig. 9A(a)). Most of interest, CD4+ T cells responded with proliferation when incubated with the l-TT_p_-l-GFP-l linker peptide (Fig. 9A(d)), but did not respond to the corresponding linker peptide l-l-GFP-l (Fig. 9A(c)) which only differs with respect to the absence of the TT_p_ sequence. Similar results were obtained for PBMCs from three other human donors (data not shown).

These data indicate (i) the artificial antigen linker module can be taken up by APCs in PBMCs, and (ii) be processed and thereby TT-related T-cell epitope(s) are released and presented by MHC class II molecules of the selected donor to CD4+ T cells resulting in a proliferation in an antigen-specific manner. Furthermore, it shows that the purified recombinant proteins are free of contaminations such as lipopolysaccharides (LPS).

### Induction of proliferation of T cells after delivery of a TT-derived T cell epitope to slanDCs via the novel immuno targeting system

Finally, we tried to show that our novel immuno targeting system can indeed be used for the delivery of an antigen to slanDCs in PBMCs and that this delivery can lead to an antigen specific response of T cells. As slanDCs are not the only APCs in PBMCs we developed the following strategy: From the presented data we knew that (i) the l-TT_p_-l-GFP-l linker peptide can be taken up at 37°C by APCs in PBMCs, (ii) but does not bind and is not taken up by slanDCs or any other APC in PBMCs if incubated at 4°C, (iii) multivalent anti-slan scBsDb/linker peptide complexes can be formed and specifically bind to slanDCs at 4°C but not to the surface of other APCs present in PBMCs, and (iv) unbound material can be removed by washing. Consequently, SL scBsDb/linker module complexes formed and bound at 4°C on the surface of slanDCs will not be removed by a washing step at 4°C while unbound material will be removed. Therefore, unbound components cannot be taken up by either other APCs present in PBMCs or slanDCs. Only, scBsDb/linker module complexes will remain and can eventually be taken up by slanDCs if the temperature is raised to 37°C after washing. In the case that the immune complexes formed on the surface of slanDCs will be eaten up by APCs after raising the temperature to 37°C but not by slanDCs then GFP fluorescence should be found within APCs others than slanDCs.

Indeed, the specific delivery of the TT_p_ T-cell epitope to slanDCs via the SL scBsDb/l- TT_p_-l-GFP-l linker complex results in a proliferation of the autologous CD4+ T cells (Fig. 9B(c)) while the single components of the complex (Fig. 9B(a,b)) are unable to induce proliferation. Similar results were obtained for PBMCs from three independent human donors (data not shown). Moreover, GFP was never seen in any other cells from PBMCs (data not shown).

Taken together, our data indicate that (i) the SL scBsDb can form a multivalent anti-slan scaffold with peptide linker modules that contain repetitive l-tags, (ii) T-cell epitopes can be inserted in these artificial linker modules, (iii) insertion of peptide epitopes does not affect the formation of the complex and the binding to slanDCs, (iv) SL scBsDb/linker modules can be used for the delivery of antigens to slanDCs, and (v) antigens bound to the slan epitope can be taken up by these cells, processed and presented on MHC class II molecules to T cells.

## Discussion

Dendritic cells are key players in the immune system in that they link the innate immune response to the adaptive immune response [15]. Most of DCs in blood and peripheral tissues are immature. In this state, they efficiently phagocytose antigens. Following interaction with antigens and concomitant activating determinants, DCs undergo a maturation process resulting in an upregulated expression of adhesion, costimulatory, and MHC molecules. Microbial products, including for example cell wall components, non-methylated CpG DNA and double stranded RNA have been identified as potent inducers of DC activation [16]. Once mature, DCs are less phagocytic, and readily migrate to the draining lymph nodes where they present the processed antigens in the context of MHC to naïve T cells leading to activated effector CD4+ helper and CD8+ cytotoxic T cells. In the conventional antigen presenting pathway, endogenously synthesised proteins including viral proteins from virus-infected cells are presented by MHC class I molecules. Antigens that are taken up e.g. by phagocytosis are directed to lysosomes where they are processed and then presented by MHC class II molecules. DCs have an alternative pathway for class I presentation known as “cross-presentation”, where cell-associated or soluble antigens access and are presented by MHC class I molecules to CD8+ T cells [17].

The progress in understanding the function of DCs has stimulated much research on DC-based vaccination strategies [18]. In the mouse model, the delivery of antigens via DCs was successfully used for both initiation of antigenic tolerance and initiation of immune responses, e.g., for reprogramming the immune system against tumor cells [19], [20]–[27].

Dendritic cell-based vaccines use autologous DCs that are either generated *in vitro* from monocytes or isolated from patients. The DCs are then loaded *ex vivo* with antigen prior to re-administration to patients. Targeting DCs *in vivo* via specific surface receptors represents a more direct and less laborious strategy.

Much was learned about *in vivo* targeting of DCs in mice. Antigens were experimentally delivered to murine DCs *in vitro* or *in vivo* via the DEC-205 receptor [20], [28]. DEC-205 is a multilectin receptor for adsorptive endocytosis that is expressed in mouse DCs and some epithelia. For delivery of antigenic material to this receptor, antigenic peptides are recombinantly fused to and expressed as parts of an antibody to the DEC-205 receptor [18], [28]–[31]. This increases the efficiency of presentation of antigens on both MHC class I and class II molecules *in vivo* [20], [21], [30]–[32]. DEC-205 was also used for immuno targeting of human DCs which were generated *in vitro* from monocytes of healthy donors and malignant melanoma patients [33]. Targeting of DCs may also be possible in humans as many types of human DCs express more or less restricted marker molecules such as langerin by Langerhans cells, DC-SIGN by dermal DCs, or BDCA2 by pDCs.

As summarized in the introduction section, slanDCs represent a major interesting subset of myeloid derived human blood DCs that is capable of inducing neoantigen-specific CD4+ T cells as well as tumor-reactive CD8+ CTLs [9], [11]. Therefore, this subset of human blood DCs represents an attractive candidate for an *in vivo* delivery of antigens for vaccination including against tumor cells [12].

SlanDCs can be stained in human PBMCs with the anti-slan IgM mabs MDC8 and DD2 [9]–[13]. However, the slan epitope does not exist on mouse DCs. In contrast to mouse DCs, slanDCs are negative for the DEC-205 epitope (Bachmann, data not shown). Consequently, human slanDCs cannot be targeted with an anti-DEC-205 antibody. The use of anti-slan IgM mabs for treatment of humans is also difficult because they cannot be sufficiently purified and lose their binding capability when monovalent derivatives are prepared (as shown herein). Moreover, it was unclear whether or not antigens delivered to the slan modification of PSGL-1 will be taken up by slanDCs, processed and presented to T cells.

Therefore, we first tried to elicit high affinity IgG-type anti-slan abs. However, in spite of a series of attempts we were not successful to establish high affinity abs to the sulfated carbohydrate structure representing the slan epitope. The final reason remains unclear. One possible explanation could be that the PSGL-backbone does not provide sufficient T cell help for class switch/somatic hypermutation. In order to overcome this problem we decided to develop a convenient modular cell targeting complex for slanDCs. The idea was to multimerise recombinant monovalent antibody derivatives (scBsDbs) via suitable linker molecules and, thereby, not only to restore the binding capability of the polyvalent maternal IgM antibodies but also to deliver antigenic peptides to slanDCs. Such antigen containing linker molecules can easily be created by multiple tagging of the desired epitope sequence(s) with the l-Tag. This procedure is much less laborious and easier than fusion and recombinant expression of whole antibody-antigen fusion proteins.

As shown in the result section we were indeed able to realise such a versatile and modular scaffold: The novel immuno targeting system allowed us to deliver a TT-derived T-cell epitope, which was apparently taken up, processed and presented by slanDCs resulting in the proliferation of CD4+ T cells. An uptake of antigens via PSGL-1 is also supported by a recent publication of Maugeri *et al* [34].

In our setting the proliferation of CD8+ T cells is hardly measurable, including in the TT-positive control, therefore, we presently don't know whether or not the T-cell epitope taken up via the slan epitope can also be cross-presented to CD8+ T cells.

For the formation of the scaffold we used in our studies linker modules containing an antigen flanked by repetitive l-Tags. For a clinical application the backbone flanking the antigen(s) should not be antigenic or an immune response to the backbone should not be critical. The l-Tag might fulfill these criteria: As mentioned above the l-Tag represents an aa sequence which is present in the C-terminal domain of the autoantigen La/SS-B. Twenty to thirty percent of patients with systemic lupus erythematosus and up to 90% of patients with Sjögren's syndrome develop antibodies to this nuclear antigen. Moreover, a varying percentage of about 5% of healthy donors can also be found positive for the La antigen [35], [36]. Anti-La antibodies are assumed as epiphenomena in these patients and even reported to be protective against anti-dsDNA autoantibodies [35], [36]. When we tested a series of anti-La positive sera (n = 25) of autoimmune patients for reactivity to the 7B6 peptide epitope sequence representing the l-Tag none of the tested anti-La positive autoimmune patient sera reacted with the 7B6 peptide epitope (data not shown). Consequently, an induction of an immune response to the 7B6 peptide epitope in humans appears unlikely as it does not even occur in autoimmune patients developing an anti-La autoimmune response. And, if accidentally induced, an anti-La response should not become life threatening as anti-La antibodies are assumed to be non-pathogenic [35] and can even occur in healthy individuals [36].

The novel modular cell targeting system is not limited to the anti-slan IgM DD2. It should also be useful for introducing other low affinity antibodies into therapy. By replacement of the anti-slan specificity with another specificity, e.g., against a tumor-associated antigen the scaffold system could be modified for either drug delivery to tumor cells or redirecting of immune effector cells to tumor cells or the improvement of tumor imaging.

In the future, even more sophisticated linkers could extend the versatility of this novel immuno targeting system.

## Materials and Methods

### Ethics statement

Peripheral blood mononuclear cells (PBMCs) were isolated from fresh blood by Ficoll-Hypaque density centrifugation as described previously e. g. [11]. Alternatively, we used buffy coat cells which were obtained from the transfusion blood center of the German red cross. The materials were used with the informed written consent of all participants. The study including the consent form was approved by the local ethics committee of the university hospital of the medical faculty of Carl-Gustav-Carus TU-Dresden (EK27022006).

### Preparation and expression of the novel SL scBsDb

For cloning of the SL scBsDb, the heavy and light chain sequences of the two mabs DD2 and 7B6 were determined. For this purpose, cDNAs were prepared from total RNAs that were isolated from the respective hybridomas using TriPure reagent (Roche, Mannheim, Germany) and the Advantage RT-for-PCR Kit (Clontech/TaKaRa, Saint-Germain-en-Laye, France). The respective cDNAs were amplified by PCR using the Advantage HF 2 PCR Kit (Clontech/TaKaRa, Saint-Germain-en-Laye, France) and primer pairs according to Wang et al. [37]. For amplification of the heavy chain of the anti-slan mab DD2 we used the primers HCfw2 (TT TTT GGA TCC SAR GTN MAG CTG SAG SAG TCW GG) and IgM rev (ATT GGG ACT AGT TTC TGC GAC AGC TGG ATT). The resulting PCR products were subcloned into pGEM-T easy (Promega, Mannheim, Germany) and sequenced. The pGEM-T clones were used as substrate for a splicing by overlapping (SOE) PCR using the primers DD2_HC SfiI fw (CCA TGG CGG ACT ACA AAG ATA TTG TGC TGA CCC AGT CTC C), DD2_HC GS rev (CCA GAA CCA CCA CCA CCA GAA CCA CCA CCA CCG GAT ACA GTT GGT GCA GCA TC), DD2_LC GS fw (TGG TGG TTC TGG CGG CGG CGG CTC CGG TGG TGG TGG ATC CGA AGT TAA GCT GGA GGA G), and DD2_LC NotI rev (GCG GCC GCG ACA TTT GGG AAG GAC TGA C) to construct the DD2 scFv (Fig. 1B).

For construction of the bispecific DD2x7B6 scBsDb (SL scBsDb, Figs. 1D, 2A), the 7B6 heavy chain was amplified with the primers HC7B6scFv 1F (GGC GCC GGC GGC TCC GGT GGT GGT GGT TCC GGT GGT GGT GGC TCC ATG GCG GAC TAC AAA GAG GTC) and HC7B6scFv 2R (GGA TCC ACC ACC ACC AGA GAC AGT GAC CAG AGT CCC TTG GC). The 7B6 light chain was amplified with the primers LC7B6scFv 1F (GGA TCC GAC ATT GTG CTC ACA CAG TCT C), and LC7B6scFv 2R (GGC GCC AGA ACC ACC ACC ACC AGA ACC ACC ACC ACC GGG CCC GGA TAC AGT TGG TGC AGC ATC AGC). The light chain was cloned upstream of the 7B6 heavy chain sequence using the artificially created *Kas*I restriction enzyme site in the linker sequence (all restriction enzymes used were purchased from Fermentas, St. Leon-Roth, Germany). This 7B6 light chain-heavy chain construct was then cloned into the DD2 scFv sequence using *Bam*HI restriction enzyme sites resulting in the SL scBsDb reading frame.

For expression of the recombinant antibodies, all scFv reading frames and also the reading frame of the SL scBsDb were subcloned into the vectors pSEC-Tag2B and pcz CFG 5.1. For this purpose artificial restriction enzyme sites (either *Sfi*I and *Not*I or *Eco*RI and *Kpn*2I) were introduced by PCR. The resulting pSEC-Tag2B constructs were used for transient transfections of HEK293T cells using Lipofectamine 2000 (Invitrogen, Karlsruhe, Germany) according to the protocol of the manufacturer. The pcz CFG 5.1 constructs were used to establish permanent cell lines by transduction as described previously [38]. Recombinant scFvs or scBsDbs were isolated from cell culture supernatants by affinity chromatography using Ni-NTA agarose (Qiagen, Hilden, Germany).

### Construction of (antigen) linker peptides

Linker peptides suitable for (i) oligomerisation of the SL scBsDb, and (ii) delivery of possible antigens to slanDCs, were cloned containing the l-Tag either two times (Fig. 1F) or three times (Fig. 1G(i) and (ii)). In all linker molecules, two of the l-Tag epitopes were separated by green fluorescent protein (GFP) which served as a marker and spacer molecule. An experimental antigen-containing linker peptide was obtained by cloning a Tetanus Toxin (TT) derived antigenic peptide (TT_p_) representing AA506–526 (NYSLDKIIVDYNLQSKITLP) of TT between the first and second l-Tag as schematically shown in Fig. 1G(ii). The different linker modules were termed l-GFP-l (Fig. 1F), l-l-GFP-l (Fig. 1G(i)), and l-TT_p_-l-GFP-l (Fig. 1G(ii)).

The cloning of the linker peptides was performed as follows: The clone pET28b_La AA311–328 which encodes the minimal 7B6 aa epitope sequence served as template for PCR using the primers LaEpitope_N(XhoI_AatII)_1F (5′- CTC GAG AAA GAA GCA CTG AAG AAA ATA ATA GAA GAC CAA CAA GAA TCC CTA AAC AAA ACT AGG GAC GTC AGT ACT GAG AAA GAA GCA CTG AAG -3′) and pET28_SmaI_4R (CCC GGG AAA ACA GCA TTC). The respective PCR fragment was cloned into pET28b_La AA311–328 creating a novel clone with two l-Tag sequences in a row separated by an artificial *Aat*II restriction enzyme site. Herein, the EGFP reading frame sequence was inserted. For cloning of the construct containing three l-Tag elements, the pET28b_l-GFP-l DNA served as template for PCR using the primers LaEpitope_N(Spe I)_2R (5′- ACT AGT TTT GTT TAG GGA TTC TTG TTG GTC TTC TAT TAT TTT CTT CAG TGC TTC TTT CTC GCT AGC CAT ATG GCT GCC GC -3′) and pET28_Bgl II_1F (GAG GAT CGA GAT CTC GAT C). Finally, this sequence was cloned into the pET28b_l-GFP-l vector DNA resulting in the pET28b_l-l-GFP-l plasmid DNA sequence. For delivery of the TT-derived antigenic peptide TT_p_ the AA sequence NYSLDKIIVDYNLQSKITLP was inserted in between the first and second l-Tag element of the pET28b_l-l-GFP-l plasmid. For this purpose pET28b_l-l-GFP-l plasmid DNA was amplified by PCR using as primers the primer TT_506–525_ (Hind III) R (5′-AAG CTT CCT ACG AGG AAG AGT AAT TTT AGA TTG AAG ATT ATA ATC AAC AAT AAT TTT ATC AAG AGA ATA ATT TCG TCT CGG ATC CCG ACC CAT TTG CTG) in combination with the primer pET28 (Mlu I) 3F (5′-AAT GAT CAG CCC ACT GAC GC). The resulting construct was termed as the pET28b_l-TT_P_-l-GFP-l. Plasmids encoding the respective linker peptide were transformed into *Escherichia coli* (*E. coli*) BL21(DE3)pLysS bacteria (Merck-Novagen, Nottingham, UK). Expressed recombinant proteins were purified from bacterial extracts by Ni-NTA agarose affinity chromatography under native conditions and analysed by SDS-PAGE/immunoblotting. Detection was performed with either anti-penta-HIS antibody (Qiagen, Hilden, Germany) or the anti-La mab 7B6 and anti-mouse IgG conjugated with alkaline phosphatase (Dianova, Hamburg, Germany).

### Cell culture, FACS analysis and epifluorescence microscopy

Commercially available Jurkat cells (ATCC TIB-152) were grown in RPMI medium (Invitrogen, Karlsruhe, Germany) containing 10% fetal calf serum (FCS, Biochrom, Berlin, Germany). Slan(+) Jurkat cells represent a subpopulation present in Jurkat cells (ATCC TIB-152): According to previous observations [11] about 5 to 10% of Jurkat cells express the slan (previously termed MDC-8) epitope. Such slan(+) Jurkat cells can be enriched from Jurkat cells using magnetic anti-IgM beads (Miltenyi Biotech, Bergisch-Gladbach, Germany) coated with the anti-slan mab MDC8 as described previously [11], [13] (see also Fig. 4A(a, red graph). SlanDCs were isolated from PBMCs using the same approach.

For FACS analysis, slan(+) cells were stained with either the anti-slan mab DD2 or the respective SL scBsDb/linker peptide complex. The binding of single components of the respective SL scBsDb/linker peptide complex was analysed in parallel. Briefly, cells were incubated with 50 µl of hybridoma cell culture supernatant containing the anti-slan mab DD2 and stained with a commercially available PE-conjugated anti-IgM antibody (BD Biosciences/Pharmingen, Heidelberg, Germany). For complex formation the respective linker peptide was pre-incubated for 1 h at 4°C with the SL scBsDb. Formed SL scBsDb/peptide linker complexes were incubated for an additional 30 min to 1 h with either slan(+) Jurkat cells, isolated slanDCs or PBMCs. Bound complexes were detected with an Alexa488-labeled anti-penta-HIS mab (Qiagen, Hilden, Germany). In the case of slanDCs, PE-conjugated anti-CD16 and PE-Cy5-conjugated anti-HLA-DR were added to all samples. For FACS analysis HLA-DR-positive monocytes were gated and slan(+) cells were plotted versus CD16-positive cells. The amount of double positive cells is the percentage of total cells. Data analysis was performed using WinMDI 2.8 software.

Stained cells were documented by epifluorescence microscopy using a Zeiss Axiovert 200 M epifluorescence microscope and the Axio vision software package (release 4.6.3, Zeiss, Jena, Germany).

In the case that the GFP fluorescence present in the linker modules was not sufficient for detection it was enhanced by staining with an anti-GFP antibody covalently linked with FITC (Acris Antibodies GmbH, Herford, Germany).

If not noted otherwise, all stainings were performed for 1 h at 4°C.

### Proliferation assay

For the analysis of proliferation, isolated PBMCs were labelled with carboxyfluorescein succinimidyl ester (CFSE) according to a protocol described by Mannering *et al*. [39] using 0.5 µM CFSE in PBS. Afterwards, cells were resuspended in medium and incubated in the absence or presence of indicated proteins (1×10^6^ cells/well in 2 ml medium in 24-well plates) for 8 days. TT (Behringwerke, Marburg, Germany) was used at a concentration of 5 µg/ml, linker peptides were used at a concentration of 350 nM. Alternatively, 1.4×10^6^ cells/well were pre-incubated with recombinant proteins or pre-formed protein complexes (0.7 nmol l-TT_p_ -l-GFP-l linker peptide, 2.8 nmol SL scBsDb) for 1 h at 4°C in a total volume of 400 µl in 24-well plates. Afterwards, cells were washed twice with PBS to remove unbound proteins and pellets were resuspended in 2 ml of medium. Medium used for culturing of CFSE-labelled PBMCs consisted of RPMI 1640 (Biochrom, Berlin, Germany) supplemented with 2 mM L-glutamine, 1 mM sodium pyruvate, 1% (v/v) nonessential amino acids, 100 µg/ml penicillin, 100 µg/ml streptomycin (all from Biochrom, Berlin, Germany) and 10% (v/v) human serum (CC pro, Neustadt, Germany).

Cells were cultured for 8 days and stained with PE-conjugated anti-CD3 and PE-Cy5-conjugated anti-CD4 (BD Biosciences/Pharmingen, Heidelberg, Germany) and analysed by flow cytometry. CD3-positive cells were used to display CFSE versus CD4 staining.
